# StripeRust-Pocket: A Mobile-Based Deep Learning Application for Efficient Disease Severity Assessment of Wheat Stripe Rust

**DOI:** 10.34133/plantphenomics.0201

**Published:** 2024-07-23

**Authors:** Weizhen Liu, Yuxi Chen, Zhaoxin Lu, Xiaoyu Lu, Ze Wu, Ziyao Zheng, Yongqiang Suo, Caixia Lan, Xiaohui Yuan

**Affiliations:** ^1^ School of Computer Science and Artificial Intelligence, Wuhan University of Technology, Wuhan, Hubei 430070, China.; ^2^ Wuhan University of Technology Chongqing Research Institute, Chongqing 401120, China.; ^3^ Sanya Science and Education Innovation Park of Wuhan University of Technology, Sanya, Hainan 572025, China.; ^4^ Hubei Hongshan Laboratory, College of Plant Science and Technology, Huazhong Agricultural University, Wuhan 430070, Hubei, China.; ^5^ Engineering Research Centre of Chinese Ministry of Education for Edible and Medicinal Fungi, Jilin Agricultural University, Changchun, Jilin 130118, China.

## Abstract

Wheat stripe rust poses a marked threat to global wheat production. Accurate and effective disease severity assessments are crucial for disease resistance breeding and timely management of field diseases. In this study, we propose a practical solution using mobile-based deep learning and model-assisted labeling. StripeRust-Pocket, a user-friendly mobile application developed based on deep learning models, accurately quantifies disease severity in wheat stripe rust leaf images, even under complex backgrounds. Additionally, StripeRust-Pocket facilitates image acquisition, result storage, organization, and sharing. The underlying model employed by StripeRust-Pocket, called StripeRustNet, is a balanced lightweight 2-stage model. The first stage utilizes MobileNetV2-DeepLabV3+ for leaf segmentation, followed by ResNet50-DeepLabV3+ in the second stage for lesion segmentation. Disease severity is estimated by calculating the ratio of the lesion pixel area to the leaf pixel area. StripeRustNet achieves 98.65% mean intersection over union (MIoU) for leaf segmentation and 86.08% MIoU for lesion segmentation. Validation using an additional 100 field images demonstrated a mean correlation of over 0.964 with 3 expert visual scores. To address the challenges in manual labeling, we introduce a 2-stage labeling pipeline that combines model-assisted labeling, manual correction, and spatial complementarity. We apply this pipeline to our self-collected dataset, reducing the annotation time from 20 min to 3 min per image. Our method provides an efficient and practical solution for wheat stripe rust severity assessments, empowering wheat breeders and pathologists to implement timely disease management. It also demonstrates how to address the “last mile” challenge of applying computer vision technology to plant phenomics.

## Introduction

Wheat stripe rust, caused by *Puccinia striiformis* f. sp. *tritici* (*Pst*), is a destructive foliar disease that poses substantial challenges to wheat production worldwide [1,2]. This disease, also known as yellow rust, is characterized by bright yellow to orange uredinial pustules on wheat leaves, forming long, narrow, and stitching-shaped lesions along leaf veins [2]. Disease severity, quantified as the proportion of lesion areas on the leaf blade, serves as a widely adopted trait for scoring wheat stripe rust. It provides an estimation of pathogen infection severity and plant susceptibility [3]. Traditionally, wheat breeders and pathologists conduct field evaluations by visually scoring disease severity for each wheat variety. This approach is not only labor-intensive and time-consuming but also prone to fatigue-induced errors and inconsistency, especially when dealing with a large number of plants. Accurate and efficient disease severity assessment technologies are urgently needed to alleviate the tedious and repetitive field work of researchers.

Image-based methods for assessing the disease severity of plant leaves have received widespread attention [4–8]. Accurately segmenting disease lesions, leaves, and backgrounds from leaf images is a crucial step. In the early days, several image processing-based algorithms, such as the adaptive threshold-based Otsu method, were proposed for symptomatic area segmentation and severity assessment [9–16]. These approaches typically require the manual selection of informative features associated with diseases in images, such as color, texture, and edges, and their segmentation performances are strongly dependent on specialized handcrafted extractors. Later, machine learning algorithms were investigated for the segmentation of lesion areas on soybeans, oranges, and other plants [17,18]. Using random forest and noise reduction spatial processing, Heineck et al. [19] reported a segmentation pipeline that quantifies pustule numbers and estimates disease severity for crown and stem rusts on perennial ryegrass. However, the performance of this method is not very satisfactory because of the poor generalizability of datasets with diverse image shooting conditions and complex backgrounds.

In recent years, deep learning (DL) algorithms have exhibited remarkable performances in semantic segmentation, making considerable progress in the segmentation of wheat stripe rust. Li et al. [20] proposed a U-Net-based model to segment stripe rust lesion areas from wheat leaf images under diverse illumination and angles in a greenhouse setting, achieving a mean intersection over union (MIoU) of 83.44%. However, their evaluation of complex background leaf images still lacks precision. To address this issue, recent research has focused on 2-stage segmentation algorithms, aiming to improve the accuracy of segmentation. Wang et al. [21] developed a 2-stage DL model called DUNet, which integrates DeepLabV3+ and U-Net for quantifying cucumber leaf disease severity in complex backgrounds. The initial segmentation step uses the DeepLabV3+ model to separate the leaf from the surrounding background, allowing for a more precise analysis of the leaf area. In the second stage, the U-Net model further identifies and distinguishes the lesion and healthy leaf areas within the segmented leaf portion. By dividing the leaf into these specific regions, the model can accurately determine the extent and location of the lesions and distinguish them from healthy leaf tissue. Divyanth et al. [22] also generated a similar 2-stage model for measuring leaf diseases in corn field imagery. Despite all recent successes, one major drawback of these algorithms is that they are computationally intensive and rely on powerful computing resources. This hinders their direct deployment on resource-constrained handheld devices such as smartphones or tablets, which are commonly used in field evaluations.

With the advancement of smartphones, there have been mobile applications (APPs) incorporating lightweight networks in the field of agriculture. For instance, Sherafati et al. [23] developed an Android APP named TomatoScan, which utilizes a multilayer perceptron network to predict the ripening stages of tomatoes. Rimon et al. [24] developed PlantBuddy, a server-based Android APP that detects plant diseases by accessing a MobileNetV2 network on the server side. To predict changes in the coloration of citrus fruit peels, Bao et al [25]. employed a lightweight variant of the U-Net network to develop an offline Android APP. This method exhibits commendable scalability and can be readily extended to different citrus species and other fruit crops. However, these approaches rely on sacrificing model accuracy. Currently, there is a lack of algorithms that strike a balance between lightweight design and accuracy, particularly for offline DL-based assessment of plant disease severity on mobile devices. In fact, plant disease severity is one of the most critical disease traits for conducting field surveys.

Additionally, one major challenge in developing DL algorithms is the requirement for a substantial quantity of labeled data [26]. The pixel-level manual annotation process for constructing semantic segmentation-based algorithms is extremely labor-intensive and costly. This challenge is particularly pronounced in the agricultural domain, where precise labeling of specific categories often requires specialized expertise [27]. Unfortunately, the availability of these experts is limited. To overcome these challenges, many efficient labeling strategies have been studied to reduce the manual effort required from annotators while maintaining annotation quality. Model-assisted labeling is one such strategy that incorporates existing models trained on similar or related tasks into the labeling workflow. For instance, artificial general intelligence (AGI) models such as the segment anything model (SAM) [28] have been incorporated into automated or interactive labeling tools. Unfortunately, due to the challenges involved in annotating agricultural domains, the labeling results from these tools often fail to meet the requirements of precise annotation. Therefore, Zhu et al. [29] designed a model-assisted annotation pipeline for wheat epidermal cells, markedly reducing the annotation time from approximately 1 h per image to only 10 min. Transfer learning is another effective labeling strategy widely adopted in agriculture [30]. By utilizing the weights of pretrained models from large-scale datasets such as ImageNet as initial settings, transfer learning enables the transfer of learned knowledge and features to specific agricultural tasks, reducing the annotation workload and enhancing the performance of DL models [31]. Nevertheless, to the best of our knowledge, there is currently no efficient labeling pipeline for plant disease segmentation annotations.

Overall, the aim of this study is to improve disease severity assessment technology for wheat stripe rust by providing a practical, user-friendly, and consistent method involving model-assisted labeling, mobile-based DL, and mobile APP development. To be more precise, the main contributions are summarized as follows.

1. User-Friendly Mobile APP: We introduce an Android-based offline mobile APP named StripeRust-Pocket, which directly deploys the proposed StripeRustNet algorithm. The APP provides an intuitive interface that allows users to easily capture images of wheat stripe rust leaves, obtain on-site disease severity assessment results, organize results into an Excel format, and send out an Excel spreadsheet via email. These functionalities enhance the practicality and adoption of disease assessment techniques in real-world agricultural settings.
2. Stripe Rust Dataset with an Efficient Labeling Pipeline: We provide a dataset of wheat stripe rust images consisting of 5,013 images with a resolution of 3,472 × 4,624 pixels. To ensure efficient and high-quality annotation, we develop a 2-stage labeling pipeline. This pipeline has significant advantages compared to automatic or interactive labeling tools. It incorporates model-assisted labeling, manual correction, and spatial complementarity strategies. By applying this approach to annotate 642 selected images for model training, the average annotation time was significantly reduced from 20 min to 3 min.
3. Improved Disease Severity Assessment Model: We propose a 2-stage balanced lightweight network architecture that combines MobileNetV2-DeeplabV3+ and ResNet50-DeepLabV3+ for assessing disease severity in wheat stripe rust leaf images under complex backgrounds. This design achieves a balanced trade-off between segmentation accuracy and computational efficiency.

## Materials and Methods

### Dataset

In April 2021, at the Ezhou Research Station of Huazhong Agricultural University in Hubei, China, we collected our own dataset. The dataset consists of 5,013 high-resolution images with a resolution of 3,472 × 4,624 pixels. Smartphones, including Xiaomi K40, Xiaomi MIX2, an iPhone 12, and an iPhone 12 Pro, were used for image acquisition. The images were captured at shooting distances between 15 and 20 cm under various sunlight illuminations and shooting angles. To ensure a uniform background, we placed a plastic whiteboard under the leaf of interest and captured the image from above the leaf. The dataset is publicly accessible. From our own dataset, we selected 642 images for model training. Additionally, to increase the complexity of the background, we included 82 images from a public wheat leaf dataset sourced from Kaggle [32] for model training. The Kaggle dataset comprises 208 images of wheat leaves affected by stripe rust, captured at Holeta Wheat Farm in Ethiopia using a digital single-lens reflex camera (Canon EOS 5D Mark III).

In summary, we selected a total of 724 images from the 2 datasets, representing a balanced distribution of different backgrounds and disease severity levels captured under field conditions. Image examples are shown in Fig. S1. Among these, 624 images were used to construct a disease severity quantification model based on a 2-stage semantic segmentation approach. The remaining 100 images were reserved for testing the final 2-stage disease severity quantification model.

### Data annotation

**Fig. 1.**
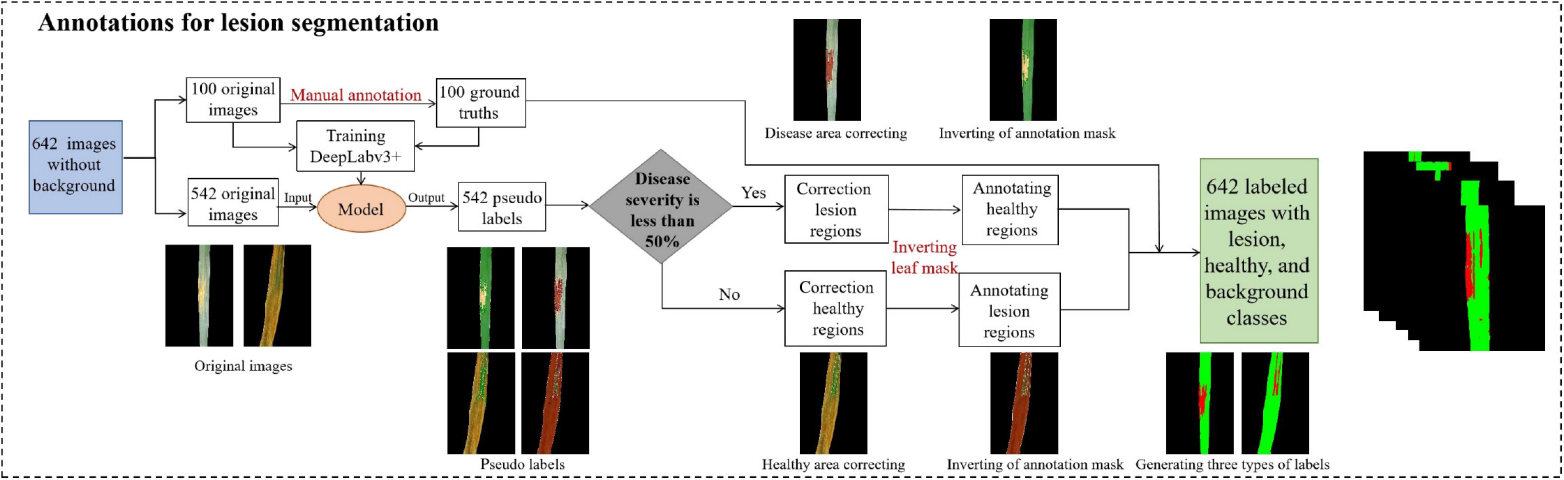
The semiautomatic annotation pipeline for lesion segmentation.

An annotation pipeline was developed for label generation to meet the requirements of a 2-stage segmentation network. Initially, the leaves are separated from the backdrop through manual annotation techniques, which facilitates the acquisition of imagery devoid of background. Given that the pixelwise segmentation annotations of lesion regions are particularly labor-intensive and time-consuming, a number of strategies have been devised for workload mitigation. The comprehensive annotation method, along with the respective masks, is depicted in Fig. 1. The annotation pipeline proposed herein proved considerably more efficacious than the conventional manual approach. An average of approximately 3 min was necessary to produce an annotation with manual refinement, in stark contrast to the 20 min required for a fully manual annotation per image.

#### Annotations for lesion segmentation

This step involved labeling lesions and healthy regions in the extracted leaf portion. After removing the background, the resulting images contained only lesions and healthy pixels. A model-assisted labeling method was utilized to create labels. The process involved the following steps: (a) Manual annotation: A total of 100 images were manually annotated using the LabelMe tool [33], which allows users to annotate objects in images by drawing bounding boxes or polygons around the leaf. (b) Model training: The annotated images were then used to train the DeepLabv3+ model for 200 epochs. During training, image augmentation techniques such as flipping, rotation, shifting, and scaling were applied to enhance the generalization ability of the model. (c) Prelabel Generation: Subsequent to the training phase of the model, the residual dataset comprising 542 images was input into the trained network to engender a pair of masks for each respective image. These generated masks are designated prelabels. The dual prelabels procured for every image represent the diseased and healthy regions. (d) Manual correction: In pursuit of authentic annotations, the prelabels underwent meticulous manual adjustments utilizing the LabelMe annotation tool. To optimize the annotation efficiency, we manually corrected the easier class and used spatial complementarity to generate annotations for the other class. As shown in Fig. 1, for wheat leaves with low stripe rust severity (disease severity ≤50%), the lesion parts were manually annotated, while for leaves with high severity, the healthy parts were manually labeled. A total of 642 images with 3 categories of labels, including lesion, healthy, and background, were obtained.

### Data augmentation

In real-world applications, the leaf exhibits various orientations and positions in the acquired image, which affects the robustness of the semantic segmentation-based disease severity quantification model. To address this issue, the following geometric transformations were randomly applied to each image in the training dataset of the leaf segmentation network and the lesion segmentation network using Albumentation [34] as the data augmentation library. The horizontal flip method is one of the most commonly used data augmentation methods. Therefore, we implemented the horizontal flip operation to mirror the image along its vertical axis. Rotation was restricted within the range of −90° to 90°. Additionally, for direct transformations of the image, we applied shifting to its height and width. The shifting range was limited to −0.0625 to 0.0625. Finally, to simulate the variability in pixel sizes observed in real-world scenarios, we scaled the pixel dimensions of the images to between 80% and 120% of their original size. This adjustment was made to better reflect the diverse pixel resolutions encountered in the captured images.

### StripeRustNet: 2-stage model for disease severity assessment

#### Overview of the StripeRustNet workflow

**Fig. 2.**
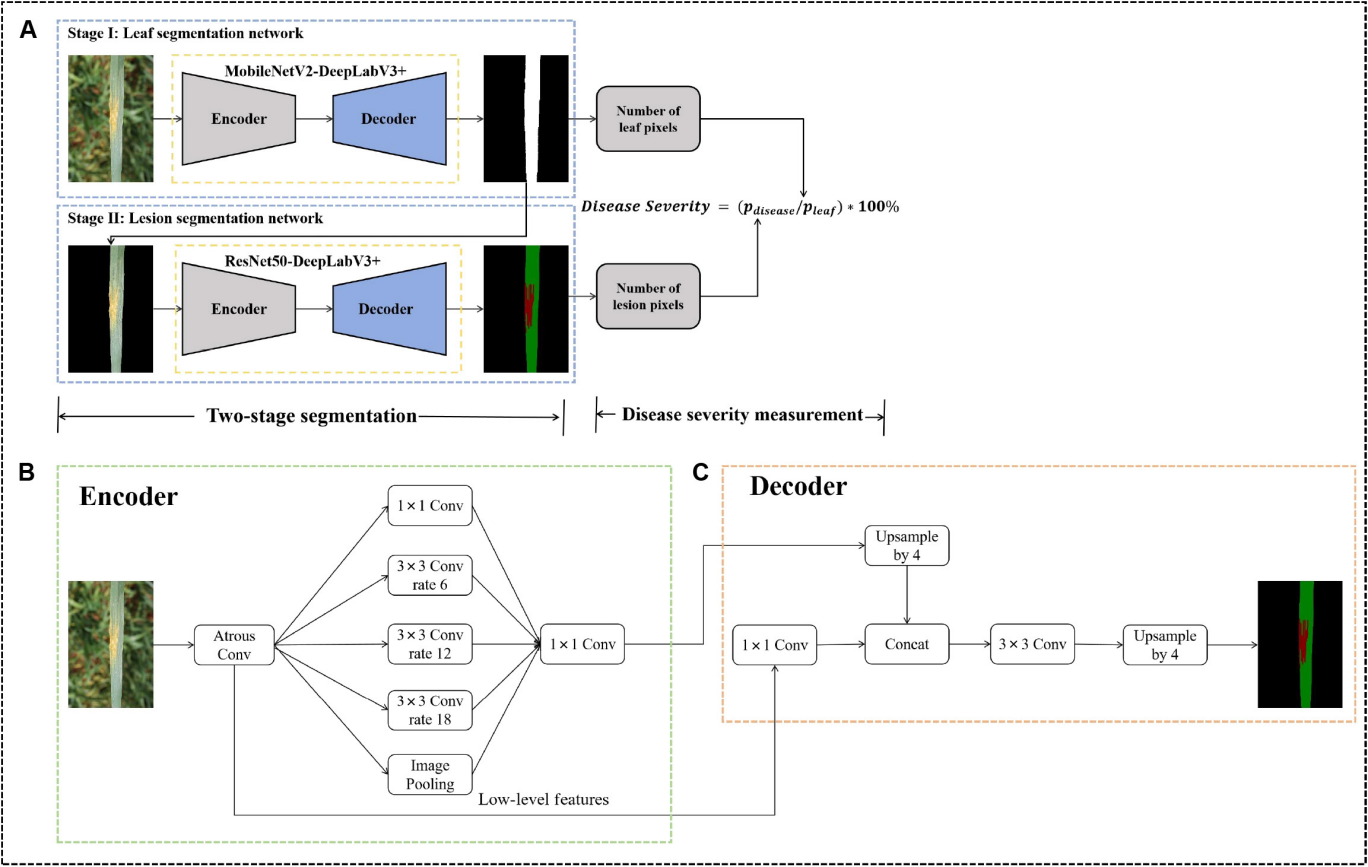
The pipeline architecture of StripeRustNet for quantifying disease severity in wheat stripe rust. (A) The overall architecture of StripeRustNet. (B) The detailed architecture of the DeepLabv3+ encoder. (C) The detailed architecture of the DeepLabv3+ decoder.

Accurate segmentation of leaf and stripe rust lesions is crucial for accurate assessment of disease severity. However, the one-stage segmentation model struggles to segment the leaf and lesion together in complex backgrounds, resulting in low segmentation accuracy. In contrast, the 2-stage segmentation model, with its dual classification approach, faces less difficulty in segmenting either the background and leaf or the leaf and lesions in each round. To make the proposed algorithm more computationally efficient, a 2-stage model integrating MobileNetV2-DeeplabV3+ and ResNet-DeepLabV3+ was developed as the segmentation model for this study. The disease severity trait is calculated by quantifying the ratio of the total pixel count of the lesion spots to the pixel number of the leaf blade area, utilizing the obtained segmentation results. The overall architecture of the proposed disease severity assessment model, called StripeRustNet, is illustrated in Fig. 2.

#### Two-stage balanced lightweight segmentation model

To identify the most suitable segmentation model, a comprehensive evaluation was conducted on 4 commonly used CNN-based networks: DeepLabV3+ [35], UNet [36], PSPNet [37], and DANet [38]. After considering segmentation performance, model parameter size, and inference time, DeepLabV3+ emerged as the top-performing model for both stages. Consequently, StripRustNet is based on DeeplabV3+.

DeeplabV3+ [35] is a lightweight architecture designed for semantic segmentation tasks, which utilizes techniques such as atrous convolution and depthwise separable convolution to reduce parameters and computational complexity. The network architecture of DeepLabV3+ is shown in Fig. 2. The choice of backbone has a substantial impact on the speed and accuracy of the model, and DeeplabV3+ exhibits high compatibility with different backbones, allowing us to select suitable backbones for different tasks in the 2-stage pipeline. In the first stage, considering the relatively simple shapes of wheat leaves, segmentation is relatively easier. Therefore, we selected MobileNetV2 as the backbone network [39]. MobileNetV2 utilizes the inverted residual structure, where inverted residuals are composed of 2 1 × 1 convolutions and a 3 × 3 depthwise separable convolution. Additionally, MobileNetV2 employs the linear bottleneck technique, which replaces the Rectified Linear Unit (ReLU) activation function of the last 1 × 1 convolutional layer with a linear activation function. These techniques enable MobileNetV2 to substantially reduce computational costs and memory usage while maximizing model accuracy.

In the second stage, when the shapes and sizes of stripe rust lesions are more complex, the segmentation task becomes more challenging. Hence, we adopted ResNet50 as the backbone network. ResNet50 adopts the ReLU activation function and is primarily composed of 4 residual blocks. Each block consists of 2 1 × 1 convolutions and 1 3 × 3 convolution. The 4 residual blocks are organized into groups of 3, 4, 6, and 3 blocks. Notably, the second and third residual blocks have strides of 2, while the remaining 2 blocks have strides of 1. Additionally, before entering the residual blocks, ResNet50 begins with a 7 × 7 convolutional layer with a stride of 2. It concludes with a fully connected layer. As a result, ResNet50 is a deeper network capable of capturing more intricate and fine-grained features, which is crucial for accurately segmenting diseased regions from leaf images [40].

The 2 DeepLabV3+ models were integrated in the following manner. Initially, the first-stage MobileNetV2-DeepLabV3+ model was utilized to perform inference on the input image, resulting in a mask image that captured the initial leaf segmentation. This leaf mask image was temporarily saved for further processing. Subsequently, the original image was cropped based on the generated leaf mask image to obtain a pure leaf image with the background removed. The pixel count of the leaf (*p_leaf_*) was then calculated using this extracted leaf image. The obtained leaf image was further processed by the second-stage ResNet50-DeepLabV3+ model for inference, generating a mask image that delineates both the lesion and healthy regions. Additionally, the pixel count of the lesion region (*p_lesion_*) was determined. Finally, the second-stage model produced a segmented result image, and the disease severity trait was calculated using the following equation:

$$\text{Disease severity}=\frac{p_{\text{lesion}}}{p_{\text{leaf}}}\times 100\%$$ (1)

#### Loss function

The cross-entropy was employed as the loss function of the proposed StripeRustNet. Cross-entropy loss is a distribution-based loss that is widely applied for classification and segmentation tasks [41]. It measures the dissimilarity between the ground-truth distribution and the predicted distribution. For the DL-based semantic segmentation task, the cross-entropy loss function can be formalized as:

$$\begin{array}{c} L_{\mathrm{CE}}\left(y,p\right)=-\frac{1}{N}\sum_{n=1}^{N}\sum_{c=1}^{C}y_{n,c}\text{log}p_{n,c} \end{array}$$ (2)

where *C* represents the number of classes, *N* represents the number of pixels, *y*_*n*,*c*_ ∈ {0, 1} represents the ground truth, and *p*_*n*,*c*_ ∈ {0, 1} represents the predicted pixel class.

### Transfer learning-based network training

Given the limited size of our dataset, training a highly effective neural network from scratch poses considerable challenges. To overcome this limitation, we adopted transfer learning, a technique that leverages pretrained models from other image segmentation tasks as a starting point [31]. In this study, we initialized the weights of our backbone networks, MobileNetV2 and ResNet50, with pretrained weights from the ImageNet dataset [42]. This initialization provided a strong foundation for our models to learn and adapt to the specific features of the wheat stripe rust severity assessment tasks.

The hardware used to train and test the StripeRustNet model is a graphics processing unit (GPU) server equipped with an Intel Xeon (R) e5-2650 CPU, 11 GB of memory, and 4 GeForce GTX 1080ti GPUs but only 2 of the 4 GPUs were used. The entire training process is implemented using the PyTorch framework running on the CentOS 7.7 operating system. The SGD optimizer is adopted. The batch size is set to 4. The initial learning rate of the encoder is 0.01. The learning rate of the decoder is initially set to 0.001 and gradually decreases to 0 according to the poly strategy, whose equation is as follows:

$$\mathrm{lr}=lr_{0}*{\left(1-\frac{i}{T_{i}}\right)}^{\text{power}},\text{power}=0.9$$ (3)

The entire training process spans 200 epochs, during which the image dataset is expanded using online augmentation. This augmentation method focuses on the “batch” level, wherein various transformations are applied to the input image and its corresponding labels synchronously during training, thereby increasing the diversity of the image samples. Consequently, the number of trained images can increase as the number of training iterations increases.

In this study, each batch of data was initially resized to a fixed size of 640 × 640 before being input into StripeRustNet. The image dataset was randomly divided into training, validation, and testing sets at a ratio of 6:2:2. For the training set, a series of geometric transformations were uniformly applied for online data augmentation. These transformations included a horizontal flip with a probability of 0.5 and a random rotation within the range of 0° to 90°.

### Evaluation metrics

The evaluation metrics used to evaluate the performance of StripeRustNet are as follows. The pixel accuracy (PA) is defined as the proportion of the correctly predicted pixels to the total number of pixels with the following formula:

$$\mathrm{PA}=\frac{\mathrm{TP}+\mathrm{TN}}{\mathrm{TP}+\mathrm{FP}+\mathrm{FN}+\mathrm{TN}}$$ (4)

where true positive (TP) is the number of pixels that are accurately predicted to be the given class, true negative (TN) is the number of pixels that are accurately predicted to not belong to the given class, false positive (FP) is the number of pixels that are not the given class but are predicted to be the given class, and false negative (FN) is the number of pixels that are the given class but are predicted to not belong to the given class.

The intersection over union rate (IoU), also known as the Jaccard index, is one of the most well-recognized indicators of success for semantic segmentation tasks; it considers scale invariance and perceptual qualities [43]. For each class, the IoU metric is defined as the ratio of the number of correctly classified pixels to the total number of true-positive, false-negative, and false-positive pixels. In this study, we calculated the IoUs for the lesion, healthy, and background classes, referred to as the IoU-L, IoU-B, and IoU-H, respectively. The IoU equation is as follows:

$$\mathrm{IoU}=\frac{\mathrm{TP}}{\mathrm{TP}+\mathrm{FN}+\mathrm{FP}}$$ (5)

The MIoU is the most popular evaluation metric for semantic segmentation tasks [44]. It averages the IoU across all the classes, which is calculated as:

$$\begin{array}{c} \text{MIoU}=\frac{1}{k+1}\sum_{i=0}^{k}\frac{p_{\mathrm{ii}}}{\sum_{j=0}^{k}\mathrm{pij}+\sum_{j=0}^{k}p_{\mathrm{ji}}-p_{\mathrm{ii}}} \end{array}$$ (6)

where *k* + 1 is the number of pixel classes with *k* foreground classes and backgrounds, *p_ii_* denotes the number of pixels that are both predicted and labeled as class *i*, and *p_ij_* denotes the total number of pixels that are predicted as class *i* but labeled as class *j*.

The inference time of a single image is a commonly used indicator to evaluate the inference speed of algorithms. To measure accurately, a “GPU warm-up” process is performed by making 50 predictions using the model. This initializes the GPU and prevents it from entering the power-saving mode during the time measurement. To ensure a precise inference time computation and avoid incorrect measurements caused by other tasks preempting the GPU cores, the PyTorch script *synchronize* was employed. It ensures that the task stream on the current GPU core is fully completed before measuring the inference time. The final inference time of a single image is reported as an average of 300 measurements.

### Design for the Android platform

#### Deployment of the StripeRustNet model

The StripRustNet model cannot be directly deployed on Android devices. To address this issue, PyTorch Android Lite was utilized for model deployment. PyTorch Android Lite [45] is a set of tools that enables DL model deployment on smartphones, facilitating DL inference on mobile devices. Its primary functionalities include model format conversion and optimization, reducing the computational complexity of the model and improving the performance and efficiency of mobile devices. First, 2 script traces and the optimize_for_mobile function of PyTorch were used to transform the Python model into the pt (PyTorch Serialized Tensor) format and the pt model into the ptl (PyTorch Serialized Tensor Lite) model. Subsequently, the ptl model could be loaded onto smartphones for DL inference.

After obtaining the ptl-format model, loading this model on an Android device is a crucial step. We need to know the complete storage path of the ptl model and utilize the functionality provided by the PyTorch Android Lite module to load the model based on the path. Additionally, to successfully perform image inference, it is necessary to convert the images stored in the Android system in 2-dimensional bitmap format into one-dimensional tensor-format images. Subsequently, the images are input into the model for inference, resulting in a tensor array with scores for each pixel. A loop traversal is then conducted to analyze each pixel, and the selected pixels are saved in an array with different categories represented by distinct colors. To visualize the result, a new bitmap is created to extract data from the saved collection, and the final graphical representation is drawn by assigning values to each pixel point. As we have a 2-stage segmentation model, the generated image is directly passed into the second-stage model. The aforementioned steps are repeated to obtain the final segmentation result and the disease severity of the wheat leaves.

#### APP development

**Fig. 3.**
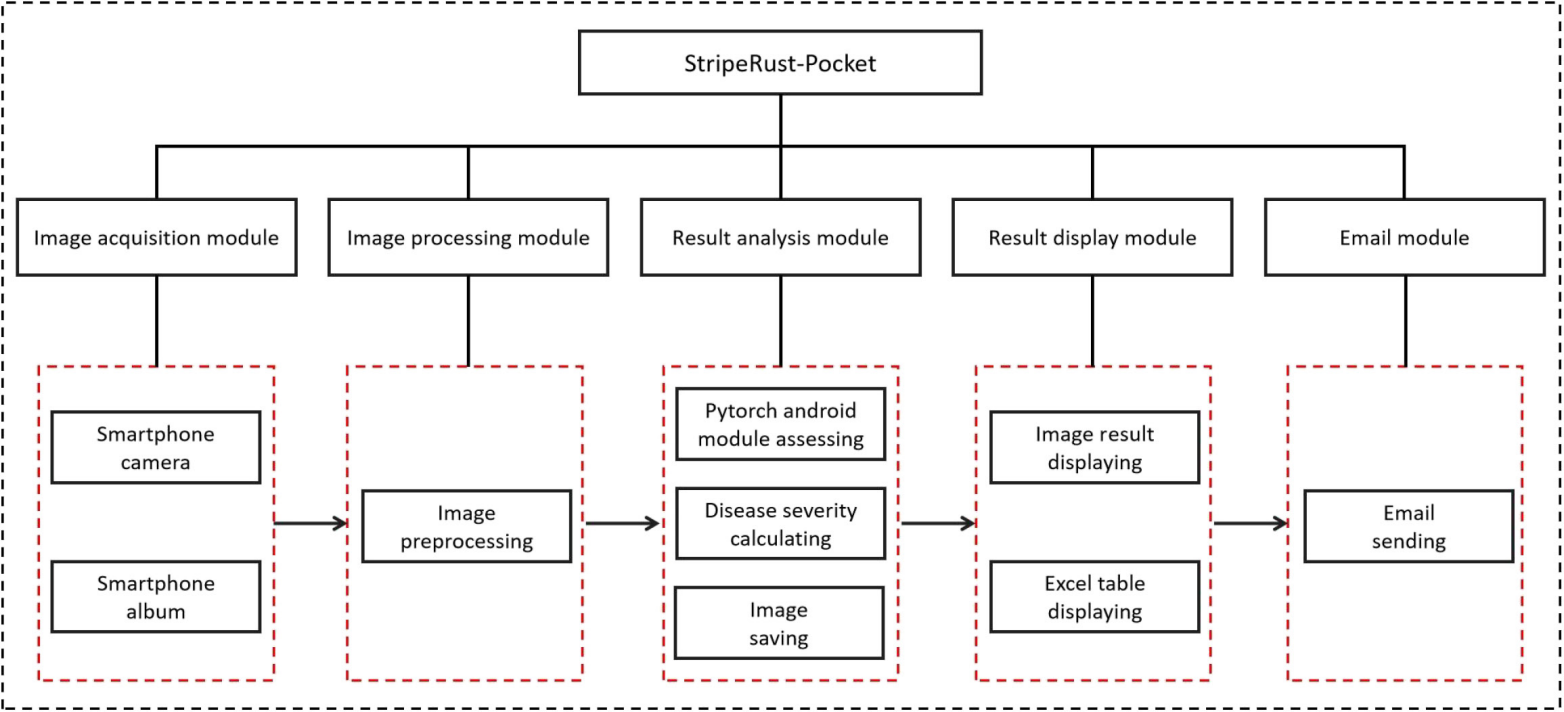
The overall structure of the StripeRust-Pocket.

We developed a mobile APP called StripeRust-Pocket, which incorporates our StripeRustNet model, to create a user-friendly phenotyping platform. The APP comprises 5 modules: the image acquisition module, image segmentation module, disease severity quantification module, result display module, and result export module, as depicted in Fig. 3.

The image acquisition module utilizes the built-in album and camera call functions in Android Studio, allowing users to select images from their phone gallery or to capture images directly using the camera. In the image segmentation module, the input images are resized to either 1,024 × 800 or 800 × 1,024, ensuring efficient image segmentation while maintaining segmentation accuracy as much as possible. The compressed images are then converted to Tensor format and fed into the PyTorch Lite model [45], resulting in the desired mask image with the lesion, healthy leaf, and background classes. The disease severity quantification module counts the number of lesion pixels and leaf pixels in the generated mask image and calculates its disease severity value. We designed a pop-up window allowing the user to enter the sample ID and date of the analyzed leaf image. These details were saved along with the visual results of the lesion segmentation and the disease severity result using the SQLite database [46]. The result display module exhibits the aforementioned information for each leaf image in a list format using ListView [47]. Additionally, we implemented click events on the display box in ListView, allowing users to open pop-up windows for modifying and deleting image information. These modifications are synchronized with the SQLite database [46]. SmartTable [48] was used to retrieve the record of each analyzed leaf image in the SQLite database to generate an Excel spreadsheet. The result export module calls the built-in email function of the smartphone and sends the resulting Excel spreadsheet as an attachment to the email sent by the user.

## Results

### Development of a smartphone APP:StripeRust-Pocket

**Fig. 4.**
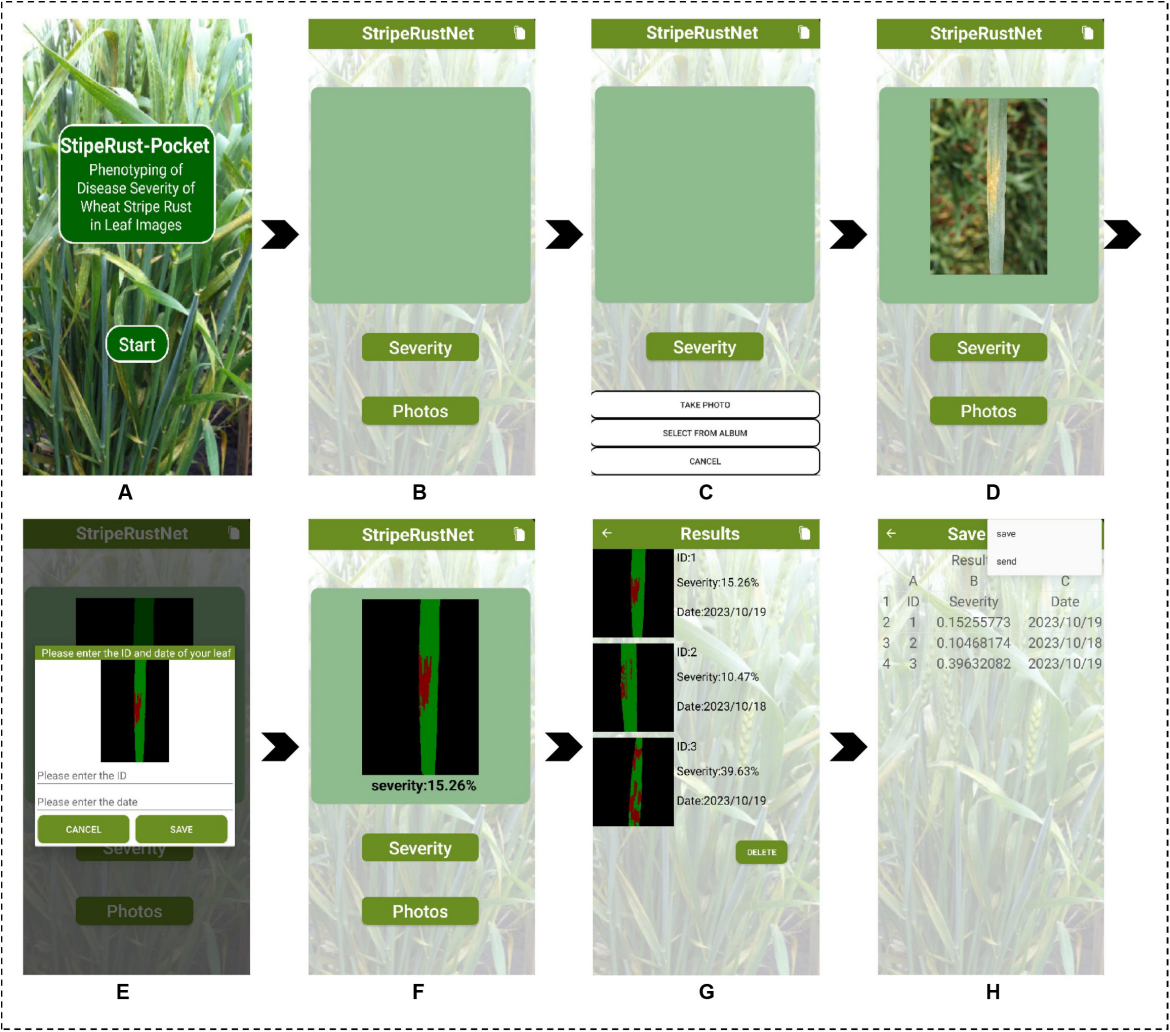
Main interfaces of the StripeRust-Pocket. (A) The startup page of the APP. (B) The main page of the APP. (C) Two options for users. (D) The image display interface. (E) The pop-up window. (F) The single result display interface. (G) The results display page. (H) The Excel page.

In this study, we developed an Android-based mobile APP to construct a user-friendly phenotyping platform. The main interface of the APP is shown in Fig. 4. Figure 4A displays the startup page of the APP, which initializes the various modules and loads the StripeRustNet DL model. After clicking the “Start” button, users are guided to the main page, as shown in Fig. 4B and C. The main page consists of a central display area and 3 buttons: “Photos”, “Severity”, and a toggle button in the top left corner. By clicking the “Photos” button, a selection box will pop up, allowing users to choose to capture images using the built-in camera or select images directly from their phone gallery. The selected image is displayed in the central display area, as shown in Fig. 4D. Subsequently, users can click the “Severity” button to perform model image inference. Figure 4E shows a pop-up window after the completion of image inference. Users can enter the result image’s ID and date and save it on their phone. After clicking the save button, the segmentation result will also be displayed on the main page (Fig. 4F). By clicking the toggle button in the top right corner, users can switch to the result display page (Fig. 4G). The result display page presents the visual outcome of lesion segmentation, user-defined ID, and date, and the final disease severity assessment for the tested image. Additionally, users can modify or delete the ID and date for each result by clicking on the respective result (Fig. 4G). By clicking the white button in the top right corner again, users can navigate to the Excel page (Fig. 4H). This page initially displays all the tested image’s disease severity results in the form of an Excel spreadsheet. Moreover, the generated spreadsheet can be directly sent via email by clicking the button in the top right corner, facilitating result analysis and sharing.

### Performance evaluation of leaf andlesion segmentation

**Fig. 5.**
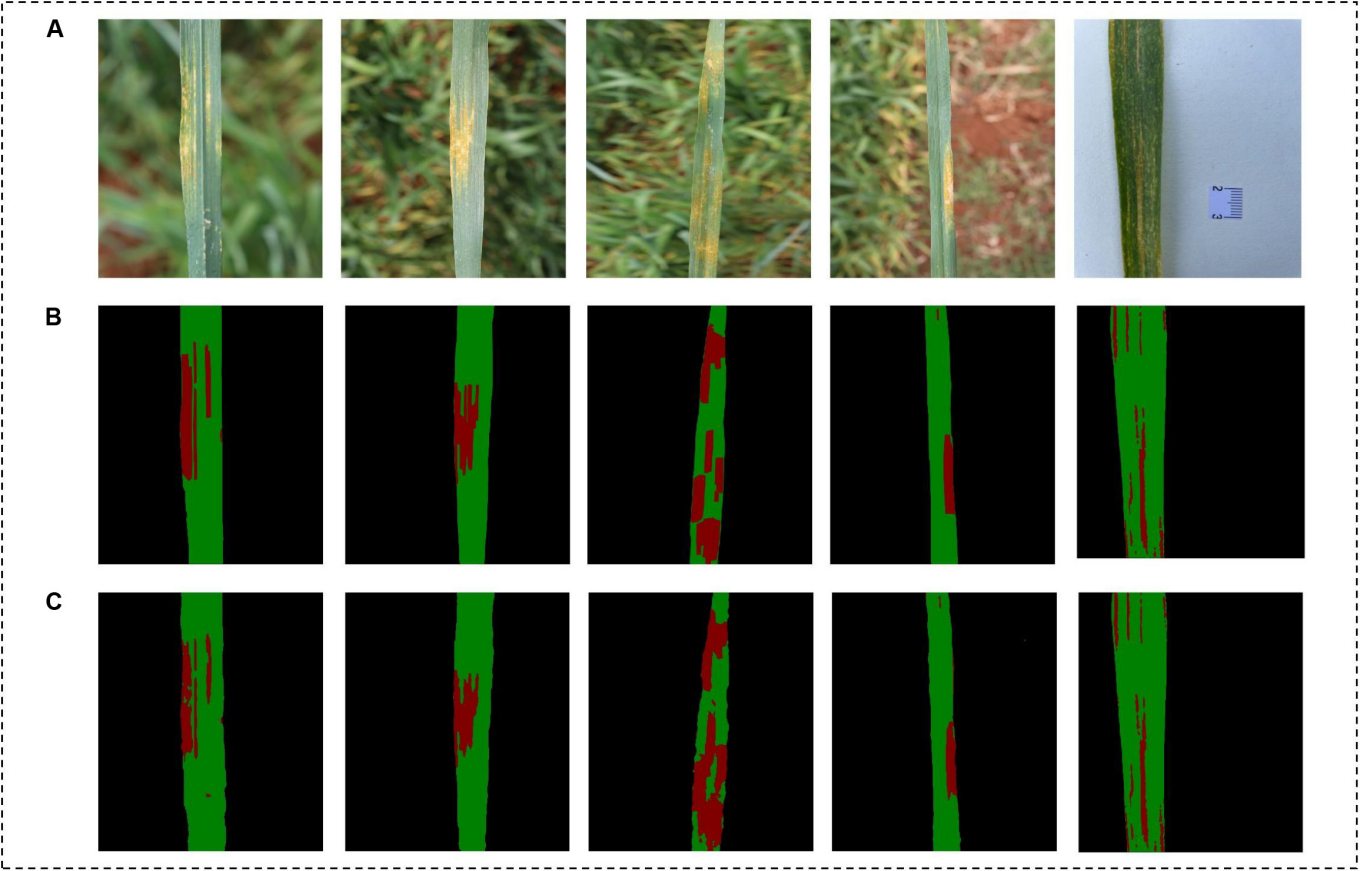
Visualization results of StripeRustNet. (A) Original images, (B) ground truth, and (C) segmentation results.

This section focuses on comparing the performance of DeepLabV3+ with that of other state-of-the-art models, namely, U-Net, PSPNet, and DANet, across 2 segmentation stages. The weight file with the best training effect is automatically saved and utilized for testing. During testing, the mask is extracted from the original image to obtain the segmentation results. The models are then comprehensively evaluated based on various metrics, including the PA and IoU for individual pixel categories, the MIoU, the single-image inference time, and the number of model parameters in both stages. The training and validation losses are presented in Fig. S2. The evaluation is conducted on the test set of 128 images, and the results for the first and second stages are presented in Table 1 and Fig. 5, respectively.

**Table 1.** Comparisons of leaf segmentation performance between DeepLabV3+ and U-Net, PSPNet, and DANet in the first and second stages. PA stands for pixel accuracy. IoU-B denotes the intersection over union rate (IoU) of the background class. IoU-Leaf denotes the IoU of the leaf class. Params denotes the number of parameters of the model. The boldface represents the optimal value in each column.

	Model	PA (%)	IoU-B (%)	IoU-Leaf (%)	MIoU (%)		Params (M)	Inference time (ms)
First stage	U-Net	99.30	99.31	97.45	98.38		26.35	120.66
	PSPNet	98.95	99.18	97.10	98.14		51.42	29.74
	DANet	99.50	**99.36**	97.47	98.42		47.56	21.39
	DeepLabV3+ with MobileNetV2 (StripeRustNet)	**99.51**	99.20	**98.10**	**98.65**		**7.6**	**13.25**
Second stage	Model	PA (%)	IoU-B (%)	IoU-H (%)	IoU-Lesion (%)	MIoU (%)	Params (M)	Inference time (ms)
	U-Net	**97.79**	**99.71**	77.34	74.06	83.70	**26.35**	120.66
	PSPNet	89.56	99.22	74.33	72.28	81.98	51.42	29.74
	DANet	97.78	99.18	74.45	73.31	82.32	47.56	**21.39**
	DeepLabV3+ with ResNet50 (StripeRustNet)	97.48	99.65	**83.53**	**75.03**	**86.08**	40.60	35.82

In the second stage, Table 1 reveals that the accuracy of the 4 models employed for lesion segmentation in the second stage is lower than that achieved in the first stage for leaf segmentation. This observation is reasonable considering the greater complexity in the shape and size of stripe rust lesions compared to those of wheat leaves. The intricate nature of these lesions poses additional challenges, making the segmentation task more difficult and resulting in lower accuracy scores. Among the 4 models, ResNet50-DeepLabV3+ demonstrated higher segmentation accuracy than did the other 3 models for lesion segmentation. It achieved IoU scores of 83.53% for the healthy category (IoU-H), 75.03% for the lesion category (IoU-Lesion), and an MIoU of 86.08%. Compared to the second-ranking U-Net model, our DeepLabV3+ model achieved improvements of 6.19% in the IoU-H metric, 0.97% in the IoU-Lesion metric, and 2.38% in the MIoU metric. These enhancements can be attributed to the deeper architecture of ResNet50. In terms of inference time and the number of model parameters, DeepLabV3+ with the ResNet50 backbone did not exhibit significant advantages over the U-Net, PSPNet, and DANet models. However, it achieved the best balance between segmentation accuracy, inference time, and the number of parameters.

In the first stage, as depicted in Table 1, MobileNetV2-DeepLabV3+ exhibited superior performance compared to the other 3 models for leaf segmentation. It achieved remarkable results, with the highest IoU for the leaf category (IoU-Leaf) and MIoU scores of 98.10% and 98.65%, respectively. Additionally, it demonstrated notable advantages in terms of inference time (13.25 ms) and number of model parameters (7.6 M), verifying its suitability for deployment on resource-constrained mobile devices. Meanwhile, we can see that the other 3 models also have sufficient segmentation accuracy. The IoU-Leaf scores for U-Net, PSPNet, and DANet are 97.45%, 97.10%, and 97.47%, respectively. This confirms our assumption that leaf segmentation is a relatively easy task.

The visualization results in Fig. 5 demonstrate the effectiveness of the proposed StripeRustNet model for leaf and lesion segmentation. The leaf images exhibit varying degrees of disease severity and are captured against different backgrounds, including soil, stems, leaves, and a whiteboard. Remarkably, StripeRustNet successfully segments both the leaves and the background, irrespective of the background type. Furthermore, StripeRustNet has the ability to accurately segment lesion areas, regardless of their shape, size, or color differences. Even the smaller stripe rust lesion spots are effectively identified and distinguished from the healthy areas, highlighting the model’s accuracy in capturing intricate details of the lesion regions.

To evaluate the effectiveness of our proposed 2-stage StripeRustNet model for wheat stripe rust segmentation, we conducted a comparative analysis with current mainstream one-stage segmentation algorithms (Table 2). Compared with Octave-Unet, which was customized by Li et al. [20] for wheat stripe rust segmentation, our StripeRustNet achieved 10.6% and 7.83% improvements in the IoU-Lesion and MIoU, respectively, highlighting its excellent segmentation accuracy. In comparison to other algorithms, StripeRustNet outperformed its competitors in many metrics, including the IoU-B, IoU-H, IoU-Lesion, and MIoU. Although ResNet50-DeepLabV3+, MobileNetV2-DeepLabV3+, PSPNet, and DANet have certain advantages in terms of inference time, segmentation accuracy remains our primary criterion. Hence, our proposed StripeRustNet maintains an ideal balance between accuracy and speed for deployment in the APP.

**Table 2.** Comparison of the segmentation performances of the proposed StripeRustNet model and the one-stage model. PA stands for pixel accuracy. IoU-B denotes the intersection over union rate (IoU) of the background class. IoU-H denotes the IoU of the healthy class. IoU-Lesion denotes the IoU of the lesion class. Params denotes the number of parameters of the model. The boldface represents the optimal value in each column.

Model	PA (%)	IoU-B (%)	IoU-H (%)	IoU-Lesion (%)	MIoU (%)	Params (M)	Inference time (ms)
StripeRustNet (2-stage model, ours)	**97.48**	**99.65**	**83.53**	**75.03**	**86.08**	48.20	48.68
ResNet50-DeepLabV3+(1-stage model)	95.33	97.02	70.38	65.88	77.76	40.60	36.45
MobileNetV2-DeepLabV3+ (1-stage model)	95.28	97.15	71.53	63.03	77.24	**7.6**	**13.25**
U-Net (1-stage model)	95.58	96.92	73.56	62.29	77.59	26.35	120.66
U-Net++ (1-stage model)	95.25	96.68	71.44	61.43	76.51	91.63	140.41
Octave-Unet (1-stage model)	95.46	97.05	73.27	64.43	78.25	32.50	112.59
SegNet (1-stage model)	95.24	97.07	70.74	59.83	75.88	167.80	224.62
PSPNet (1-stage model)	89.19	96.14	72.12	60.53	76.26	51.42	29.74
DANet (1-stage model)	95.42	96.89	71.96	60.78	76.54	47.56	21.39

### Accurate evaluation of disease severity using StripeRust-Pocket

To assess the performance of StripeRust-Pocket in disease severity detection using the mobile APP, we conducted an additional evaluation using a separate set of 100 images. These images were collected from genetically diverse wheat genotypes and exhibited varying degrees of stripe rust infection. Notably, these images were distinct from those used for model training and testing. To ensure accurate labeling, 3 experts independently provided visual scores for disease severity, thus minimizing potential subjective biases. The combination of these expert scores formed a robust mean value, which served as the reference for validating the prediction results generated by StripeRust-Pocket.

**Fig. 6.**
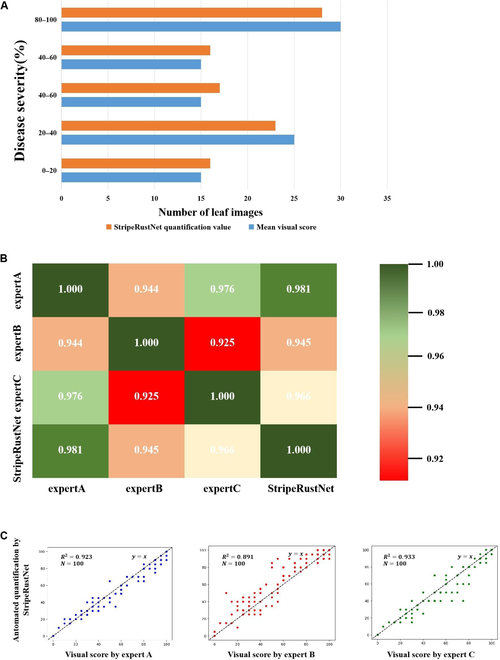
Comparison of stripe rust severity between the visual scores given by 3 experts and the automated scores quantified by StripeRust-Pocket using 100 leaf images. (A) Distributions of disease severity; (B) heatmap of pairwise correlation coefficients; (C) scatter plot and simple linear regression.

Figure 6A presents the distributions of the automated disease severity scores and the average manual scores from 3 experts. By conducting pairwise correlation analysis, we found strong correlations between the manual scores provided by each expert and the automated quantification, with correlation coefficients of 0.981, 0.945, and 0.966, respectively (Fig. 6B). Importantly, the average correlation between the automated visual score and each expert (0.964) was greater than the average correlation among the 3 experts (0.948). This suggests that the results obtained through automated quantification are more consistent and reliable than those obtained through the traditional manual approach.

Additionally, we performed simple linear regressions to fit the disease severity values provided by each expert and StripeRust-Pocket individually. Figure 6C illustrates that expert B consistently assigned lower disease severity values than did the other 2 experts and StripeRust-Pocket. This indicates the subjectivity and variability associated with classic visual scoring methods. Furthermore, we observed that the consistency of disease severity scores among the 3 experts was lower within the moderate severity range (20% to 80%) than within the very low (0% to 20%) and high (80% to 100%) severity ranges. This result reflects the inherent challenge of accurately assessing the percentage of infection in leaves with moderate infection levels.

In summary, these findings highlight the importance of developing precise and reproducible automated image-based methods for quantifying disease severity in stripe rust-infected wheat leaves. Such automated approaches effectively protect human personnel from laborious and repetitive tasks while providing more stable and reliable results.

### Speed evaluation of the StripeRust-Pocket APP

To evaluate the speed of measuring the disease severity trait using the StripeRust-Pocket APP, we performed tests on 5 different Android-based smartphones and tablets: Realme GT Neo 5, Xiaomi 13 Ultra, HUAWEI Mate 50, Realme 10+ Pro, and Huawei MatePad 11 Pro. During the testing process, 5 identical wheat leaf images at a resolution of 3,472 × 4,624 were used to ensure consistency. The results, including the average inference time per image and the corresponding standard deviation, are presented in Table 3. The tested mobile devices vary in processing chips, resulting in different measurement speeds. Among them, the Realme GT Neo 5, Xiaomi 13 Ultra, and HUAWEI Mate 50 exhibited similar performances, with inference times ranging from 5.07 s to 5.25 s per image. However, the performance of the HUAWEI MatePad 11 Pro was significantly slower, requiring twice as long as that of the fastest device, the Realme GT Neo 5. This difference can be attributed to the relatively underperforming Snapdragon 870 processor used in the HUAWEI MatePad 11 Pro. The results of the tests provide valuable insights into the performance of the StripeRust-Pocket APP on different devices, enabling us to understand the practical speed of disease severity measurement in the field.

**Table 3.** Comparison of inference time among the proposed application with different devices. SD stands for the standard deviation of inference time per image among 5 images.

Mobile device	Processor chip model	Inference time per image (s)					
		Image #1	Image #2	Image #3	Image #4	Image #5	Mean ± SD
Realme GT Neo 5	Snapdragon 8gen1+	6.44	3.3	7.19	4.14	4.27	5.07±1.48
Xiaomi 13 Ultra	Snapdragon 8gen2	5.88	3.62	7.12	4.96	4.14	5.14±1.25
HUAWEI Mate 50	Snapdragon 8gen1	6.22	3.43	7.15	5.1	4.33	5.25±1.32
Realme 10+Pro	MediaTek 1080	7.22	4.32	8.17	6.45	5.62	6.36±1.32
HUAWEI MatePad 11 Pro	Snapdragon 870	10.03	9.93	13.22	12.59	10.43	11.24±1.38

### Performance evaluation of the efficient labeling pipeline

**Fig. 7.**
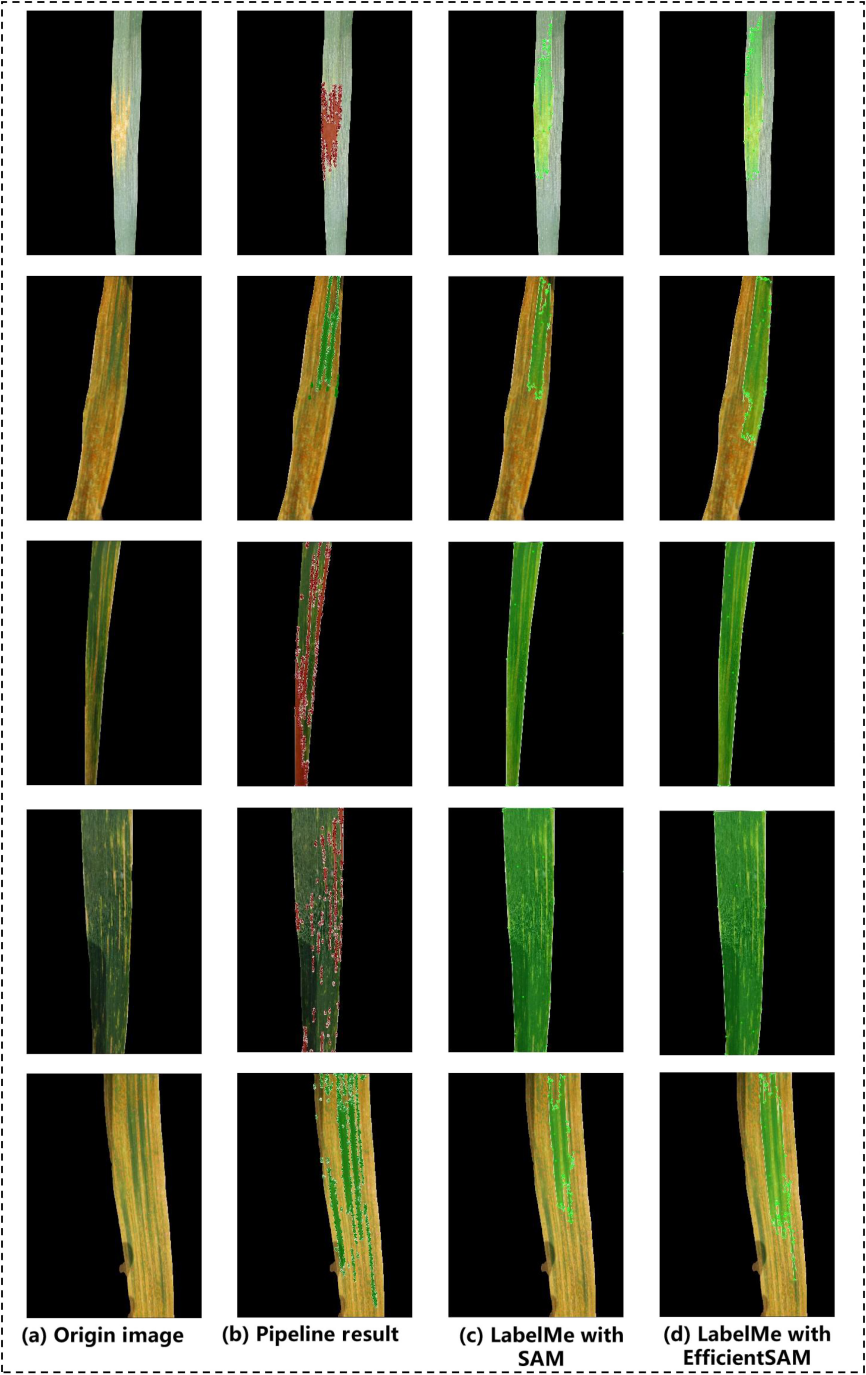
Comparison of prelabels generated by using our proposed annotation pipelines and LabelMe with different AGI models. (A) Original images; (B) annotations generated by our pipeline; (C) annotations generated by LabelMe using SAM; (D) annotations generated by LabelMe using EfficientSAM.

Currently, AGI models have been integrated into various automated or interactive labeling tools, enhancing labeling performance. This motivates us to conduct a comparative experiment between the proposed labeling pipeline and the widely used annotation tool LabelMe [33], utilizing SAM and EfficientSAM as automated annotation models. According to the visual results presented in Fig. 7, although LabelMe using SAM or EfficientSAM can roughly label relatively large lesion areas, it was not possible to accurately outline the edges of the lesions, especially small lesions. Additionally, the time cost for manually correcting the annotations generated by LabelMe was nearly equivalent to the time cost of direct manual annotation, as there were too many areas that needed to be corrected. In contrast, through our annotation pipeline, the annotation time for each image was significantly reduced from 20 min to 3 min. These results emphasize the advantages of our pipeline in annotating leaf lesion areas.

## Discussion

Disease severity plays a crucial role in assessing the impact of stripe rust infection and the susceptibility of wheat genotypes to specific *Pst* races [2]. Accurate and effective disease severity assessment is crucial for disease resistance breeding and timely management of field diseases. Despite notable advancements in computer vision techniques for plant disease quantification [4–8,20], the practical and efficient integration of these artificial intelligence (AI) tools into the plant science domain presents a significant challenge, representing the crucial “last mile” in the practical implementation of plant phenomics. To address this challenge, we developed an Android APP called StripeRust-Pocket. It enables the collection of leaf images, performs image segmentation inference, and efficiently organizes and exports disease severity data. StripeRust-Pocket is equipped with our developed segmentation DL algorithm, StripeRustNet, which accurately quantifies the ratio of lesion area to leaf area, providing an accurate phenotype for disease severity. To validate the effectiveness of StripeRustNet, we conducted extensive experiments and compared it with other segmentation DL algorithms. The results of our experiments demonstrated the superiority of StripeRustNet in terms of performance and accuracy compared to alternative algorithms. StripeRust-Pocket offers an efficient and reliable solution for disease severity assessment in wheat breeding programs and pathology research. Additionally, we introduced an efficient labeling strategy that streamlines the annotation process by leveraging techniques such as image augmentation and model-assisted labeling, reducing manual effort and improving workflow productivity.

### StripeRust-Pocket: A user-friendly phenotyping platform

Currently, there are existing image-based platforms, such as the Macrobot for powdery mildew [14] and PhenoBox for head smut fungus [49], designed to measure disease severity. However, these platforms rely on specialized hardware and imaging chambers, making them impractical and costly for individual breeding programs, and cannot be applied to field disease evaluation and control. Thus, there is an urgent need for a handheld and user-friendly disease severity data collection platform that can be used in field evaluations.

The concept of “breeder-friendly phenotyping,” introduced by Reynolds et al. [50] in 2020, emphasizes the importance of convenient, valid, readily accessible, and low-cost solutions for field phenotyping of handy traits such as foliar diseases, height, and phenology. In line with this concept, we selected smartphones and tablets as ideal candidates for constructing phenotyping platforms. These ubiquitous and mobile computing devices offer portability, high-quality imaging capabilities, and sufficient computing power. Several APPs, including PocketN [51], PocketLAI [52], PocketPlant3D [53], and PocketMaize [54], have demonstrated their effectiveness in plant image capture and analysis for phenotyping purposes.

As a response, we developed StripeRust-Pocket, an Android mobile APP dedicated to measuring plant disease severity phenotypes. Concurrently, it is a user-friendly APP that offers a streamlined user interface for data collection using a built-in camera. It directly accesses the StripeRustNet algorithm, which is deployed on the device, to facilitate efficient data processing and to visualize segmentation and disease severity measurement results. The APP is also capable of organizing and outputting disease severity data on a large scale, which greatly enhances the efficiency of field surveys for wheat breeders and pathologists assessing disease severity.

### Efficient labeling

This study presents an efficient labeling pipeline for supervised DL tasks in agriculture. By combining model-assisted labeling, manual correction, and spatial complementarity strategies in the second stage, healthy and diseased areas are labeled, and the proposed pipeline considerably decreases the workload and duration of annotation. Due to the complexity of 2-stage lesion segmentation tasks, current annotation tools, which deploy AGI models such as SAM [28], fail to meet the demand for precise labeling of diseased regions. In contrast, our annotation pipeline leverages specific models trained to achieve more accurate model-assisted labeling. Additionally, the spatial complementarity strategy enhances annotation efficiency by manually annotating diseased areas on leaves with lower disease severity and healthy areas on leaves with higher severity. This strategy capitalizes on the relative ease of annotating specific regions based on disease severity, thereby improving efficiency while preserving accuracy. Through our proposed pipeline, the annotation time per image is significantly reduced from 20 min to 3 min. This remarkable improvement in efficiency makes our annotation pipeline highly practical and scalable for disease severity assessment in real-world practice. Moreover, this approach provides valuable insights for efficient pixel-level annotation in diverse semantic segmentation tasks related to plant phenotyping. As a result, supervised DL techniques have become more accessible and feasible for agricultural APPs, enhancing their practicality and usability.

### Two-stage balanced lightweightsegmentation network

Generally, 2-stage segmentation models have advantages in terms of accuracy and complex scene processing capabilities, making them suitable for scenarios that require high segmentation results. On the other hand, one-stage segmentation models excel in inference speed and have fewer parameters, making them suitable for real-time APPs [55–57]. In our disease severity assessment task, both high-precision segmentation performance and real-time performance are required to enhance the user’s on-site experience.

To successfully deploy the 2-stage model on mobile devices with limited computational resources and achieve a balance between accuracy and efficiency, a relatively lightweight 2-stage segmentation architecture was developed. Both stages of the infrastructure utilize the lightweight DeepLabV3+ model, which provides good segmentation performance with reduced computational cost [35]. For the first stage, which involves relatively simple leaf blade segmentation tasks, lightweight MobileNetV2 was selected as the backbone network [39]. It offers fast inference speed while maintaining reasonable segmentation accuracy for this stage. However, the second stage, which involves a more complex lesion segmentation task, demands higher levels of accuracy and fine-grained feature representation. To ensure accurate segmentation, we chose to sacrifice some speed and opted for ResNet50 as the backbone network. ResNet50 has a deeper architecture and can capture more detailed features [58], leading to improved segmentation accuracy for challenging lesion segmentation tasks. By combining the lightweight DeepLabV3+ model with MobileNetV2 for the first stage and ResNet50 for the second stage, we achieved high-quality segmentation results while maintaining a promising level of responsiveness for the end user.

### Limitations and further work

The ultimate goal of the StripeRust-Pocket APP is to achieve real-time estimation of on-site disease severity. The real-time speed is typically defined as a processing rate of more than 30 frames per second (less than 33 ms per image) on a mobile device [59,60]. However, in our experiment, the StripeRust-Pocket achieved a maximum evaluation speed of 3.3 s per image on a Realme GT Neo 5 device equipped with a Snapdragon 8gen1+ processor, which does not meet real-time requirements. The processing power of smartphone chips significantly affects the inference speed [61]. Future testing on smartphones with improved processing chips is expected to further accelerate the estimation speed.

Moreover, in the future, we will focus on optimizing our mobile DL algorithms to improve the inference speed of the model. Techniques such as model compression, quantization, and pruning can be utilized to optimize DL models and reduce the computational complexity [31]. Model compression methods such as knowledge distillation or model quantization can reduce the model size without significant accuracy loss, thereby reducing memory usage and improving inference speed by minimizing memory access time [62,63]. Additionally, exploring model pruning techniques to remove redundant layers or parameters with minimal impact on performance can alleviate the computational burden and increase the inference speed [64]. While maintaining segmentation accuracy as a priority, optimizing the inference speed of StripeRust-Pocket on edge devices is crucial for achieving faster and more efficient execution of AI applications. This is particularly important for real-time measurement of disease severity in the field and for enhancing the overall user experience.

### Conclusion

Our study makes a substantial contribution to the field of wheat stripe rust severity assessment. We proposed a practical solution that leverages mobile-based DL and model-assisted labeling, addressing the challenges faced in disease severity assessments. StripeRust-Pocket is a user-friendly offline mobile APP that accurately segments diseased regions in wheat stripe rust images and determines their severity. Additionally, it serves as a portable, versatile tool for data collection, analysis, and organization. Our APP achieves an inference speed of approximately 5.1 s per image on mainstream processors. To enhance the practicality of StripeRust-Pocket, a 2-stage lightweight network architecture, StripeRustNet, was developed. This network architecture surpassed previously proposed DL-based stripe rust segmentation networks and classical semantic segmentation algorithms in terms of accuracy, inference time, and computational cost. Our model achieved an mIoU of 86.08% on the test set, with an inference time of 48.68 ms. Furthermore, we constructed a wheat stripe rust leaf dataset consisting of 5,013 images and proposed a 2-stage labeling pipeline that significantly reduced the annotation time. Our work addressed the “last mile” challenge of applying computer vision technology to plant phenomics, paving the way for advancements, and fostering interdisciplinary collaborations in the scientific research fields of breeders, plant pathologists, and others in the domain.

## Data Availability

The wheat stripe rust leaf image dataset collected by smartphones is available at https://github.com/WeizhenLiuBioinform/StripeRustNet/tree/master/Dataset. Application and source code availability: StripeRust-pocket APP: https://github.com/WeizhenLiuBioinform/StripeRustNet/tree/master/Application. The source code: https://github.com/WeizhenLiuBioinform/StripeRust-Pocket/tree/master/Application_source_code.
