# Supplementary material for: StripeRust-Pocket: A Mobile-Based Deep Learning Application for Efficient Disease Severity Assessment of Wheat Stripe Rust
Source: Plant Phenomics. 2024 Jul 23;2024:0201. doi: 10.34133/plantphenomics.0201 (PMC11265802; doi:10.34133/plantphenomics.0201)
Supplement: Supplementary 1 — Figs. S1 and S2 [file plantphenomics.0201.f1.docx]

**
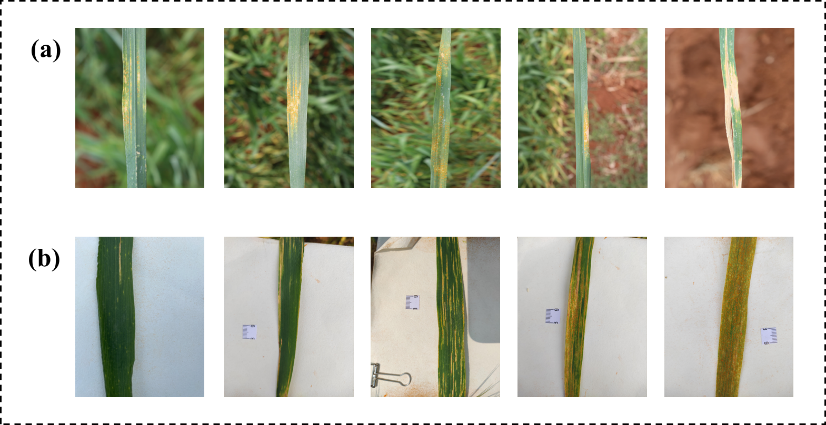
**

**Figure S1.** Sample images of our wheat stripe rust leaf dataset. (a) Images with complex field background; (b) images with the whiteboard background.


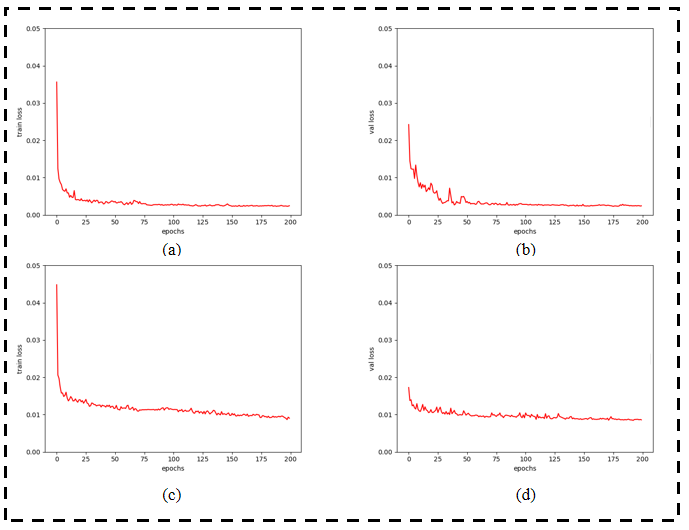


**Figure S2.** Loss curves of the StripeRustNet algorithm using CE loss functions over 200 epochs (a) Training results of the first stage; (b) validation results of the first stage. (c) Training results of the second stage; (d) validation results of the second stage.
